# Cases of Hydrophthalmia, Wherein the Practice of Arteriotomy Was Successfully Employed

**Published:** 1819-07-01

**Authors:** James Kennedy

**Affiliations:** Dunning, Perthshire


### *Cases of Hydrophthalmia, wherein the Practice of Arteriotomy was successfully employed.*

By James Kennedy, M.D. Dunning, Perthshire.

"Qui non intelligunt artes non mirantur Artifices."
C. S. A. Sidonius.

Ophthalmic dropsy is one of the most serious maladies to which the visual organ is liable; since, besides disfiguring the countenance, it too often ends in irremediable blindness, or even death itself. Various remedies have been recommended to prevent the full development of the disease; for I believe that the best occulists of the present day, consider an operation as the only means of terminating the patient's sufferings, though with the loss of sight, when hydrophthalmia is completely established. Temporal arteriotomy, however, has not hitherto, so far as I am acquainted, been announced as a therapeutic means of sufficient efficacy; and therefore, with the hope of inviting reiterated trials of its powers, I am tempted to submit the following cases to the perusal of the profession.

Case I. *August* 20*th*, 1814. A girl, ætat. 12, was this day brought to me for advice respecting her left eye. She had been employed during the summer in cow-tending, and ascribed the origin of her complaint to recent exposure to cold and moisture, in consequence of having slept with the side of her head on the ground, after a heavy shower of rain. The next day, she felt alternations of heat and shivering, with an irksome sense of constriction in the left cheek; head-ache; and painful throbbing of the temple, the integuments of which were sore to the touch. The secretion of tears was next suppressed; the eye became dry, painful, and impatient of light; all which symptoms progressively increased for some days afterwards.

The constitution of this girl evinced what might be called the lymphatic temperament. She was peevish, irascible, and the circulation easily hurried. From its extreme sensibility to light, the eye could, with difficulty, be examined. The tunica conjunctiva was red and suffused; the sclerotic vessels turgid and prominent, with a teazing sensation of foreign substances between the palpebræ and eye-ball. Through the different textures of the eye she complained of heat, itching, and lancinating pains; in short, all the phenomena which characterize severe ophthalmic inflammation were present.

Scarification of the interior palpebral surface, puncture of the angular vein; leeching, section of the temporal artery, phlebotomy, were successively *prescribed*, but no persuasions or threats could induce her to comply with any of these measures. Under existing circumstances, then, we could only exhibit an emetic, and afterwards a brisk cathartic every evening and morning for three days; applying a blister to the left temple, and another to the nape of the neck. She was placed on the lowest diet; the feet to be immersed in warm water, and the eye to be bathed occasionally with a camphorated opiate collyrium, placing an aluminous cataplasm over it in the intervals.

*August* 24*th* The patient is worse in all respects, notwithstanding the means abovementioned had acted with powerful effect. The palpebræ were greatly swollen, and could not be separated, without causing excruciating pain. The symptomatic fever was exasperated. After much trouble, this intractable girl consented to lose blood; but from the foot only. When the limb was in the warm water, the tarsal portion of the antico-tibial artery was perceived throbbing superficially; a lancet was immediately plunged into the vessel, and the blood flowed with impetuous jets, till five or six ounces were abstracted, when syncope supervened. Fomentations, poultices, pediluvia, and purgatives to be assiduously continued.

26*th.* The disease goes on uninfluenced by the means used, and acquiring daily increased malignity of character. A sensation of burning heat is now felt in the interior of the eye-ball. The headache, restlessness, irritability, and intolerance of light, are all augmented greatly. On an imperfect examination, the anterior hemisphere of the eye was discovered like one sheet of vermillion injection; the eye-lids tense and tumefied, and incapable of voluntary motion.

Scarification being now permitted, it was with difficulty performed on the conjunctive tunic, near the margin of the cornea, and procured the discharge of much extravasated and congested blood. Fomentations; anodyne embrocations; antimonials in nauseating doses; blisters; purgatives.

29*th.* The pain in the eye and neighbouring parts is partially mitigated; the eye-lids are less injected, less turgid, and less constricted; the vascularity of the sclerotic coat remarkably diminished; the retina less impatient of light. Yet the eye-ball is evidently augmented in size, and by inordinate action of the adductor muscles, it appears drawn towards the nasal angle. Its anterior chamber is enlarged; the pupil expanded, and almost incontractible; the iris seems unnaturally profound and tremulous; with a painful sense of distention in the orbit. By the girl's obstinacy we could not prevail on her to submit to bleeding, either generally or locally, nor to a seton in the neck. We could only reiterate the means before mentioned.

*September* 4*th.* Hydrophthalmia is now unequivocally established. The eye-lids are half open, and slightly reverted, while the ball, protruding in an ellipitical form from its orbit, projects about a line beyond the palpebral verges. The crystalline lens has lost its transparency, being opake and dusky; the pupil is completely dilated, and the retina nearly insensible to light. The tremulous motion of the iris is increased. The constitutional symptoms, however, are nearly the same.

My ideas of this disease induced me to urge the necessity of submitting to an evacuation of arterial blood from the vicinity of the organ affected, as the only means of preventing total blindness, and great deformity of the face. This last argument immediately removed all difficulties, and the temporal artery was divided in its ascent by the zigomatic arch. It bled furiously, and soon relieved the feeling of distention by which the patient had been distressed. After the loss of sixteen ounces, vertigo, and ultimately syncope supervened, when the operation was finished. Exposure of the diseased eye to the action of light, and to the atmospheric air was now enjoined, as far as was compatible with the feelings of the patient. Fomentations, and purgatives continued.

*5th.* The patient's general feelings somewhat mitigated; no change in the local phenomena. Eight ounces of blood were detracted from the temporal artery; perseverance in the former measures, with the addition of frictions of the stronger ammoniated liniment to the cervical portion of the spine and to the temple.

*9th.* The protruded eye-ball has nearly retired within the palpebræ, which are less tumefied and irritable; some degree of contractility observable in the pupil; the iris less tremulous. Opened the artery and bled to six ounces. Other measures to be continued.

*10th.* The local symptoms nearly the same; the constitutional disturbance nearly subsided. Six ounces of blood from the temporal artery; other treatment continued.

*16th.* During the last five days, the eye has gradually resumed its natural size and position. The patient afterwards passed through a favourable convalescence, and regained perfect health, with the exception of some degree of strabismus and dimness of vision.

Case II. *April 22d,* 1815. A peasant, in the prime of life, this day made application to me on account of a diseased eye. He stated, that sixteen days previously, he had been employed in strewing lime over a ploughed field, and that much of the calcareous particles had been blown into his eyes, mouth, and ears, by irregular gusts of wind. In the evening he complained of an aching pain in his head, accompanied with dryness, heat, itching, and distention of the eye-balls, and sensation of foreign bodies in the eye. The night was spent without sleep, and the next day, all the symptoms were aggravated, with general disquietude; alternations of heat and cold; inappetency; thirst, and soreness of the epicranial integuments—in short, acute ophthalmia of the severest form, became developed, and did not subside until after ten days of empirical treatment. On the extinction of the inflammatory symptoms, the left eye resumed its natural condition; but the right eye remained painful, with its motions embarrassed, and its vision obscured. At length the pain became excruciating, and the eye-ball rapidly enlarged, prolapsed beyond the eye-lids, lost its lustre and function of sight totally. At this time he applied to me, complaining of a distressing sense of distension in the eye and orbit, with acute pains darting through the malar, temporal, and frontal regions. He had evidently constitutional symptoms of excitement and irritation.

Ocular dropsy now existed unequivocally, and I determined on the same plan as stated in the preceding case. The right temporal artery was punctured, and jetted forth blood with great force: Twenty ounces of blood were abstracted before any of the painful symptoms became mitigated; and the loss of twenty more, removed all sense of distention, and of pain from the eye and contiguous parts. A brisk cathartic was ordered at bed-time, and to be repeated next morning.

*23d.* The purgative medicine had operated well; yet the night was passed without sleep, and the local and constitutional symptoms, with the exception of pain, had nearly resumed their previous level. Ten ounces of blood were therefore abstracted from the temporal artery, and the purgatives continued.

*25th.* No diminution of the protrusion, nor increase of visual power; but the patient thinks himself comfortable in his exemption from pain. Six ounces of blood from the temporal artery, and he was desired to keep the diseased eye bare, unless the exposure to air gave pain.

*March 5th.* The protrusion of the eye has experienced a progressive declension, assisted solely by the aid of purgatives, since last report; and with the exception of blindness, the hydrophthalmic symptoms have nearly disappeared. The man would no longer conform to medical regimen, and engaged in agricultural labours towards the end of the month. After the lapse of four years, the eye now exhibits a genuine instance of amaurosis, complicated in a slight degree with ectropium, and its concomitant, an inordinate secretion of tears.

Case III. *September 24th,* 1817. C. F. a miller, middle-aged and robust, called on me this day. He stated that, about nineteen months previously, his attention was drawn towards the state of the right eye, which felt embarrassed in its motions, with perception of extraneous bodies irritating its coverings. The following phenomena then came gradually forward; viz. Inordinate secretion of tears; tumefaction of the eye-lids; occasional inflammations of the eye; head-aches; injection of the vessels of the cornea, with distressing sense of distension in the orbit; defective vision; deep seated pain in the eye; enlargement and protrusion of the eye-ball; absolute blindness; and finally, well-marked HYDROPHTHALMIA.

At this time the globe of the eye projected more than three lines beyond the palpebræ, in a somewhat elliptic form. There was considerable eversion of the inferior eye-lid, with excoriation, and constant oozing of an irritating fluid. The pupil was dilated, and inobedient to the stimulus of light; the iris sunk, and tremulous; the crystalline lens of a reddish brown colour, and without transparency; the retina insensible to even a concentrated light.

It is needless to state the various empirical remedies that this man had used with the view of mitigating his sufferings, though without success. Still his constitution did not appear to have experienced any material detriment from the protracted irritation. The right temporal artery was punctured, and thirty ounces of blood suddenly withdrawn, when he turned sick, and, at his earnest desire, the effusion was stopped. He was directed to expose his eye freely to the action of light and air; to immerse his feet in warm water every night; to take two of the common quicksilver pills at bed-time, and, the next morning, a solution of the sulphate of magnesia.

*25th.* On inspecting the blood, the urine, and the fæces, nothing morbid could be perceived. The patient had a long and refreshing sleep last night; oppression was less felt in the head and affected orbit; the lacrymal secretion had ceased to be profuse; the other local symptoms the same. Forty ounces of blood were abstracted from the right temporal artery, without the least tendency to fainting or nausea. The pulse, however, nearly disappeared from the wrist.

*29th.* Gradually, for two days after the last bleeding, the pain in the eye increased till it became almost insupportable, accompanied by a sense of distension as though the eye-ball were ready to burst. Yesterday, however, the organ was, in a great measure, relieved from distress, and a diminution of its size was quite evident. This morning the decrease of volume is still more obvious in the eye, with collapse of its various textures. He was merely advised to guard the eye from external injuries, and to keep up a proper action in the bowels by laxative medicines.

The patient went on in slow, but favourable progress towards recovery, till the 12th of October, when it was deemed advisable to open the temporal artery, and abstract twelve ounces of blood. Thenceforward the course of recovery experienced no interruption, and by the middle of November, the eye had regained its natural proportions and position.

The sequelæ or consequences of this hydrophthalmia have been amaurosis, some degree of strabismus, epiphora, with a tumid, somewhat everted, and vascular state of the palpebræ, subjecting them to occasional excoriations consecutive of any accidental inflammatory excitement. The inconveniences, however, arising out of such a condition have not hitherto been such as to induce the patient to apply for remedial aid.

I might here, were it necessary, relate the particulars of another case, which has lately come under my observation, where temporal arteriotomy proved decidedly successful in the removal of ocular dropsy. It was a lad whose eye sustained a stroke from the horn of a bullock, and after suffering repeated attacks of inflammation, came to be greatly enlarged, and, in fact, the seat of dropsical effusion. Depletion of arterial blood, to the extent of twelve ounces, was four times performed, which, with the aid of purgatives, procured absorption of the effusedlymph from the chambers of the eye, and freed the organ from Hydrophthalmia. It remains sightless, however, and the cause of some disfigurement and annoyance, from a periodical recurrence of itchiness, and those glutinous incrustations by which psoraphthalmic affections are characterized.

Dunning in Strath-Earne, Sept. 26, 1818.

The press of important communications flowing in, deprives us of the means of giving insertion to the very ingenious train of pathological and therapeutical observations on dropsical effusions in general, which Dr. Kennedy has appended to the above interesting cases. The observations are in perfect unison with our own sentiments and experience; and derive great support from the multiplied facts brought forward in Dr. Crampton's reports on dropsy, in the present number of the Journal. Other facts and observations will shortly appear in our pages, illustrative of the sound pathological principles of Drs. Crampton and Kennedy. EDIT.

